# Median Arcuate Ligament Syndrome (MALS) in Hepato-Pancreato-Biliary Surgery: A Narrative Review and Proposed Management Algorithm

**DOI:** 10.3390/jcm13092598

**Published:** 2024-04-28

**Authors:** Lawrence Y. Lu, Jacques G. Eastment, Yogeesan Sivakumaran

**Affiliations:** 1 Critical Care Research Group, The Prince Charles Hospital, Chermside, QLD 4032, Australia; 2 Faculty of Medicine, University of Queensland, Herston, QLD 4006, Australia; 3 Department of General Surgery, Princess Alexandra Hospital, Woolloongabba, QLD 4120, Australia; 4 Faculty of Medicine and Health, University of Sydney, Camperdown, NSW 2050, Australia; 5 Department of Vascular Surgery, Princess Alexandra Hospital, Woolloongabba, QLD 4120, Australia

**Keywords:** celiac trunk compression syndrome, median arcuate ligament syndrome, pancreatoduodenectomy, liver transplantation, vascular reconstruction

## Abstract

Median arcuate ligament syndrome (MALS) is an uncommon condition characterized by the compression of the celiac trunk by the median arcuate ligament. Due to the anatomical proximity to the foregut, MALS has significant implications in hepato-pancreato-biliary (HPB) surgery. It can pose complications in pancreatoduodenectomy and orthotopic liver transplantation, where the collateral arterial supply from the superior mesenteric artery is often disrupted. The estimated prevalence of MALS in HPB surgery is approximately 10%. Overall, there is consensus for a cautious approach to MALS when embarking on complex foregut surgery, with a low threshold for intraoperative median arcuate ligament release or hepatic artery reconstruction. The role of endovascular intervention in the management of MALS prior to HPB surgery continues to evolve, but more evidence is required to establish its efficacy. Recognizing the existing literature gap concerning optimal management in this population, we describe our tertiary center experience as a clinical algorithm to facilitate decision-making. Research question: What is the significance and management of median arcuate ligament syndrome in patients undergoing hepato-pancreato-biliary surgery?

## 1. Introduction

Median arcuate ligament syndrome (MALS) occurs when the celiac trunk is compressed by the median arcuate ligament (MAL). It is also closely synonymous with Dunbar syndrome, celiac axis compression syndrome, or median arcuate ligament compression. MALS can be asymptomatic or manifest as abdominal pain, nausea, vomiting, and/or weight loss [1]. The exact prevalence of MALS remains unclear, with reported rates of 33% in autopsy studies and up to 49.7% in patients with non-specific abdominal pain [2,3]. Given its anatomical implications, MALS holds particular importance in patients undergoing hepato-pancreato-biliary (HPB) procedures, especially during pancreatoduodenectomy (PD) and orthotopic liver transplant (OLT). The reported prevalence of MALS in PD and OLT is approximately 10% [4]. This narrative review aims to explore the implication of MALS in HPB surgery and the current management approach of MALS.

## 2. Etiology, Anatomy, and Diagnosis of Median Arcuate Ligament Syndrome

The MAL is a part of the diaphragmatic crura, arising from the anterolateral surface of the bodies of the upper lumbar vertebrae (L1). The fibers arch over the abdominal aorta connecting two sides of the aortic hiatus usually at the twelfth thoracic level (T12). The origin of the celiac trunk is also situated in front of the body of the twelfth thoracic vertebrae but slightly caudal to where the MAL crosses vertebrae [5]. During embryogenesis, the celiac trunk undergoes caudal migration from the seventh cervical level due to the differential growth and descent of abdominal viscera, which results in variations in arterial anatomy [6,7]. In patients with MALS, the origin of the celiac trunk is higher relative to the insertion of the MAL. This misalignment results in the compression of the proximal part of the celiac trunk. 

**Figure 1:**
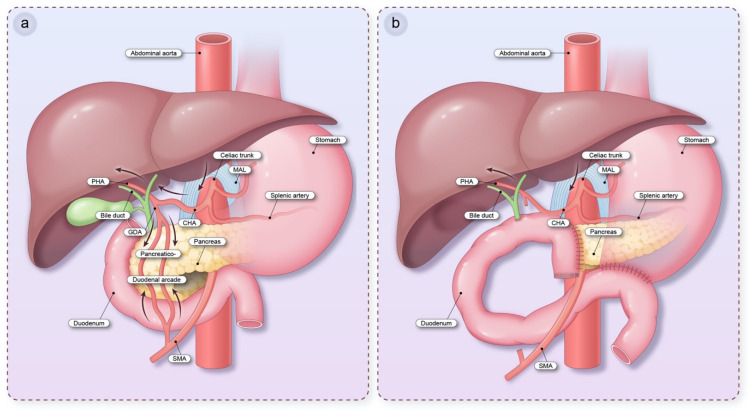
Schematic diagram of the celiac trunk. (**a**) Schematic diagram showing the celiac trunk and collateral blood supply from the superior mesenteric artery via pancreaticoduodenal arcade. (**b**) Schematic diagram showing altered blood supply after pancreaticoduodenectomy. Abbreviations: CHA, common hepatic artery; GDA, gastroduodenal artery; MAL, median arcuate ligament; PHA, proper hepatic artery; SMA, superior mesenteric artery.

In cases of MALS, blood flow from the celiac trunk is reduced, affecting its three main branches: the common hepatic artery, splenic artery, and left gastric artery. Collateral blood supply from the superior mesenteric artery (SMA) through the pancreatoduodenal arcade, gastroduodenal artery (GDA), and sometimes the distal pancreatic artery or aberrant arterial supply, helps prevent ischemia (Figure 1a). The adequacy of the hepatic collateral circulation can be assessed intraoperatively by occluding the GDA whilst either palpating the hepatic arterial flow or by using Doppler ultrasound [8]. Sugae et al. proposed a morphological grading to classify MALS into three types based on the degree of stenosis, length of stenosis, and distance from the aorta, which may be useful in predicting the likelihood of requiring treatment for MALS during primary HPB procedures [9]. However, this classification system is not sensitive and has not been clinically validated. 

MALS is typically diagnosed after an extensive workup for non-specific gastrointestinal symptoms and is often considered a diagnosis of exclusion. In patients with biliary or pancreatic pathology, it is frequently diagnosed incidentally during radiological investigations [10]. MALS can be detected using computed tomography angiography, magnetic resonance angiography, or digital subtraction angiography. It is characterized by an indentation on the superior aspect of the celiac trunk [5]. The indentation tends to alleviate during inspiration as the celiac trunk moves caudally with lung expansion and accentuates during expiration as the celiac trunk moves in the opposite direction. Therefore, mesenteric arterial duplex ultrasound is useful for the dynamic assessment of stenosis and can demonstrate elevated peak flow velocities and abnormal deflection angle [11]. In addition, while aneurysmal degeneration of pancreaticoduodenal arcades, gastroepiploic, or celiac arteries is an uncommon (~2%) complication of MALS, approximately half of the splanchnic artery aneurysms are associated with MALS [12,13,14,15]. These aneurysms carry a high risk of rupture, with mortality rates up to 30%, thus necessitating urgent embolization and division of the MAL [13,15,16].

## 3. Significance of Median Arcuate Ligament Syndrome in Hepato-Pancreato-Biliary Surgery

Pancreatoduodenectomy, commonly known as the Whipple procedure, is the standard operation for resectable cancer located within the head or uncinate process of the pancreas [17]. Less frequently, it may be indicated for conditions such as chronic pancreatitis, trauma, duodenal neoplasm, and distal biliary pathology [18,19]. The classical PD involves the resection of the head of the pancreas, duodenum, common bile duct, gallbladder, and partial gastrectomy [20]. During PD, three new anastomoses are created: hepaticojejunostomy, pancreatojejunostomy, and gastrojejunostomy [21,22]. The success of these new anastomoses relies on adequate blood supply from the hepatic artery, splenic artery, and left gastric and gastroepiploic arteries.

Foregut ischemia is not seen in MALS as there is typically a chronic and compensatory collateral circulation arising from the SMA and its branches. However, in patients undergoing PD, vessels supplying the pancreaticoduodenal arcade are routinely sacrificed with the duodenum and head of the pancreas, indicating that the blood supply for the new anastomoses then relies solely on the celiac trunk (Figure 1b). In patients with concomitant MALS, this can result in subsequent downstream ischemia postoperatively [8]. The major concern with this from a surgical perspective is the implication of local ischemia increasing the risk of anastomotic leak. Additionally, in patients with MALS, the SMA may be the dominant supply to the liver, and if this is not recognized pre-operatively, routine sacrifice of the GDA can result in devastating hepatic ischemia [23].

The contemporary indications for liver transplantation are expanding beyond the traditional pathologies of acute and chronic liver failure. Many units are also offering transplants to patients suffering from malignant pathologies, including cholangiocarcinoma, colorectal liver metastases, and hepatocellular carcinoma [24,25,26]. OLT is the approach of choice, wherein the recipient’s native liver is removed and replaced by the donor liver in the same anatomical position. The most commonly used technique for arterial reconstruction involves end-to-end anastomosis at the level of the common hepatic artery [27,28].

MALS is considered an anatomical abnormality that impedes adequate hepatic arterial flow. Similar to PD, dissection of the recipient hepatic artery often divides the collateral circulations, particularly the GDA, thereby making the celiac trunk the sole arterial supply to the liver graft. Reduced blood flow predisposes the graft to hepatic arterial thrombosis, a dreadful complication that can lead to cholangitis, poor graft function, graft loss, and death [29]. The prevalence of MALS during OLT is reported to be up to 10%, and contemporary well-powered studies on its management are scarce in the literature [30,31,32,33]. Gialamas et al. demonstrated no statistically significant difference in graft survival or post-operative biliary complications between patients with and without MALS in their retrospective case–control study [30]. They concluded that if the arterial flow to the graft is adequately established, the choice of treating MALS might not influence the overall outcome and development of complications [30]. Another single-center European study also showed no differences in short- and long-term outcomes between OLT patients with surgically treated MALS or without MALS [31]. On the contrary, a recent study reported that untreated MALS is associated with increased biliary complications and reduced graft survival highlighting the potential importance of MALS management prior to OLT [32]. Noticeably, the decision to treat MALS during OLT is arbitrary in these studies, with varied degrees of stenosis of the celiac trunk reported. These studies also differ in arterial reconstruction techniques, management of the GDA, ischemic time, and donor and recipient characteristics, which make them less comparable. The retrospective nature of these studies also precluded the exploration of a causal relationship between MALS, MAL release, and post-operative complications.

The implications of MALS are less frequently reported in other HPB procedures, such as distal pancreatectomy and Roux-en-Y hepaticojejunostomy, as they are open or performed laparoscopically with minimal disruption to the collateral blood supply. 

## 4. Management of Median Arcuate Ligament Syndrome and Our Experience

In the general population, patients with asymptomatic MALS likely do not require aggressive management of MALS. Counseling regarding MALS symptoms and the inherent risk associated with MAL release itself is an important aspect of managing this patient population. For symptomatic patients, limited evidence is available in selecting patients for MAL release and predicting symptomatic relief [34,35]. In the context of high-risk hepatobiliary surgery, although the majority of cases are detected preoperatively and the threshold for treating MALS is generally lower, there is a lack of consensus on whether and when to manage MALS. Some surgeons opt to perform it preemptively on all patients, while others base the decision on intraoperative assessment [36,37].

Open, laparoscopic, and robotic-assisted MAL release assisted by adjunctive endovascular procedures have been described [38,39,40,41]. The principle of MAL release involves the opening of the lesser sac, division of the hepatogastric ligament, and dissection of the crura until exposing the anterior wall of the aorta. The three branches of the celiac trunk, i.e., the common hepatic artery, the left gastric artery, and the splenic artery, are isolated and used to identify the celiac trunk. The skeletonization of the celiac trunk continues until it is circumferentially free of fibrous, lymphatic, and nerve attachments. 

In the general population, laparoscopic and robotic-assisted MAL release is considered the standard surgical management for MALS. In one study, 96% of patients who underwent laparoscopic MAL release experienced immediate symptomatic relief, compared to 85% of those who underwent open surgical release [42]. The clinical outcomes and rate of complications between approaches were comparable [41,42]. Regardless of the treatment approach, intraoperative assessment of the celiac flow through an ultrasound probe or arteriography is imperative to confirm the restoration of flow following the intervention. On the other hand, stenting with a balloon-expandable stent is a useful alternative, especially when the stenosis of the celiac trunk is atherosclerotic in nature, although this occurrence is less common than extrinsic compression from the MAL. Additionally, primary endovascular stenting alone is not considered the first-line management for MALS due to factors such as the dynamic position of the celiac trunk with respiration, high-grade occlusion, the risk of dislodgement with manipulation during subsequent surgery, and the potential for dissection, fracture, or occlusion following angioplasty. Rather, stenting is an adjunct to open, robotic, or laparoscopic release or as a rescue management strategy in patients with persistent stenosis after initial surgical release [43,44]. This hybrid approach has become increasingly popular in recent years [40,43,44,45,46]. Small observational studies have shown that persistent stenosis requiring stent placement occurs in 30–40% of patients after MAL release, with satisfactory outcomes observed from the endovascular intervention [43,45]. Nevertheless, in the absence of large-scale studies, future, prospective, and interventional studies specifically investigating the role of endovascular intervention in MALS are warranted.

In the context of radical HPB surgeries, which often necessitate an open surgical approach, the management of MALS can be approached in two ways: concurrently with the HPB procedure or as a two-stage procedure, wherein celiac trunk decompression is performed before the HPB surgery [10]. Concurrent MAL release offers certain advantages, such as cost-effectiveness and avoiding the need for a second-stage procedure, which can reduce waiting time and minimize the risk associated with general anesthesia, particularly in those patients with elevated anesthetic risk profiles. However, this approach may require extensive pre-operative planning and improvisation based on intraoperative findings. On the other hand, the staged approach, where laparoscopic MAL release is performed before the HPB procedure, provides additional benefits. Firstly, it allows for the assessment of the anatomy of the celiac trunk and MAL without any prior surgical manipulation that may alter the findings. Secondly, laparoscopy permits the evaluation of intraperitoneal or hepatic metastases in patients with cancer, thus avoiding laparotomy and its associated mortality and morbidity. Thirdly, MAL release alone may not suffice in restoring hepatic blood flow in more than 20% of patients undergoing PD, mainly due to a combination of atherosclerotic changes, chronicity of MALS, or scarring of the arterial wall [10,47]. Thus, the two-stage procedure allows an opportunity to confirm the restoration of adequate arterial blood flow to the celiac trunk and its branches after MAL release. In the event of failed MAL release, additional vascular reconstruction or endovascular intervention can be planned to avoid a scenario where perfusion is not completely restored prior to major foregut resection. 

Various open approaches to arterial reconstruction have been described during PD including jejunal artery-to-GDA, middle colic artery-to-GDA, celiac trunk to hepatic artery, aorto-hepatic artery, reimplantation of the celiac trunk, and bypass grafting [9,48,49,50]. The release of MAL in OLT patients with portal hypertension carries significant risk due to the presence of gastroesophageal varices and extensive collateral blood flow between the celiac trunk and SMA [4,31,51]. In some patients with extensive collateral supplies, preservation of collateral arteries and GDA without performing MAL release may provide adequate blood flow in the hepatic artery [51]. However, if surgical release fails to restore hepatic artery flow, options for hepatic artery reconstruction will be limited. Certain techniques, such as ilio-mesenteric bypass or aorto-hepatic artery anastomosis, can be challenging and have been associated with an increased risk of hepatic artery thrombosis [4,52].

In HPB patients, experience with the hybrid approach of MAL release and endovascular stenting is limited. Patient selection, outcomes, and optimal timing between endovascular intervention and radical HPB surgery have not been thoroughly studied. In contrast to the general population, preoperative intervention poses a higher risk of stent dislodgement with more extensive surgical manipulation and more severe consequences with restenosis in patients with an upcoming HPB procedure. In addition to the concerns about the feasibility and reliability of primary endovascular stenting, the necessity of antiplatelet therapy can complicate operative planning and increase the risk of bleeding. Two groups chose to perform stenting prior to PD to ensure adequate flow of the celiac trunk without dividing MAL [53,54]. Balakrishnan et al. reported an unsuccessful attempt at upfront endovascular stenting of the celiac trunk prior to PD, leading the patient to undergo subsequent MAL release and arterial reconstruction [55]. Endovascular intervention during neoadjuvant chemotherapy has been described in another case study [10]. However, MAL release was not performed in this study due to adequate flow in the hepatic artery [10]. Patients’ recoveries in the aforementioned studies were predominantly uneventful. In patients awaiting OLT, preoperative stenting can generally be avoided, as MAL release combined with arterial reconstruction offers reduced risk and superior outcomes in the majority of cases [31]. In rare cases, post-OLT stenting may be required for patients with undetected stenosis of the celiac trunk or employed as a rescue therapy for recurrent stenosis despite previous MAL release [56,57].

**Figure 2:**
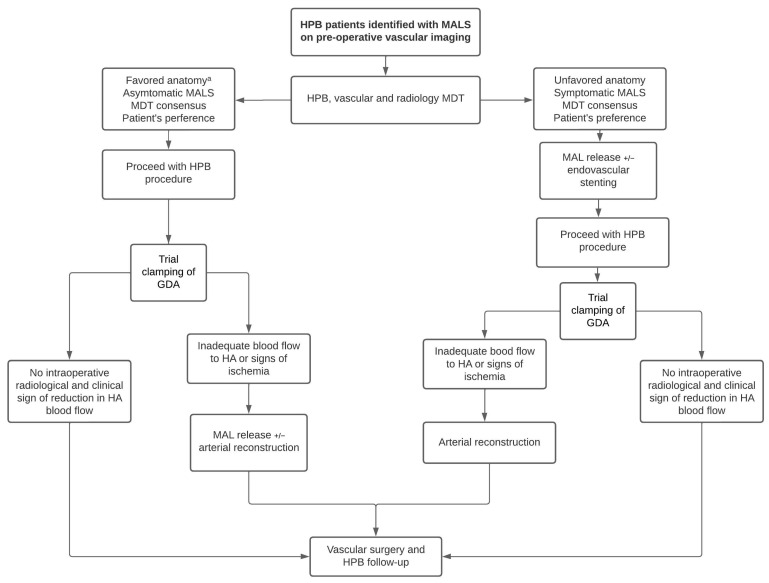
Ideal management algorithm in hepato-pancreato-biliary patient with median arcuate ligament syndrome. ^a^ favorable anatomy is defined as stenosis of the celiac trunk < 50%, preservable collateral circulation, a short segment of stenosis, and stenosis located more distal to the aorta. Abbreviations: GDA: gastroduodenal artery; HA, hepatic artery; HPB, hepato-pancreato-biliary; MAL: median arcuate ligament; MALS: median arcuate ligament syndrome.

As a tertiary hospital with interventional radiology, vascular surgery, and HPB services, our local experience highlights the importance of early multidisciplinary involvement when MALS is suspected. Drawing from the present literature review and our experiences, we have proposed a clinical algorithm for managing MALS in HPB patients (Figure 2). A multidisciplinary meeting should be convened involving radiology, vascular, and HPB surgery to determine the nature of celiac trunk stenosis (extrinsic compression vs. intrinsic stenosis), assess the need for hemodynamic evaluation on angiogram, and discuss management options while considering oncological safety or the estimated transplant date. We anticipate that patients with favorable radiological and clinical features, such as celiac trunk stenosis of <50%, a short segment of stenosis, or known asymptomatic MALS, may not require upfront intervention, as the circulation is less likely to be compromised and collateral circulation is less likely to develop [9,37]. However, it is worth mentioning that none of the mentioned features should be considered strict rules. Instead, they serve as indicators prompting surgeons to consider significant compression and a high probability of postoperative ischemia. The risk of this expectant management can be mitigated via thorough intraoperative assessment, which will be discussed later. Factors such as the chronicity and length of the stenosis, the location of the tumor, and the patient’s anatomy should also be considered. Patients should be encouraged to participate in making informed decisions and planning for their surgeries.

For patients requiring upfront treatment for MALS, which constitute a minority of cases, our preference is to perform MAL release with on-table mesenteric angiography and celiac artery stenting if required, prior to the radical surgery or transplantation, aiming to secure the hepatic arterial flow. Vascular reconstruction should be planned early, in instances of failed MAL release and stenting. In our institution, the majority of HPB patients with MALS will undergo concurrent MAL release and HPB procedures, recognizing that many cases of MALS have uncertain clinical and anatomical significance due to extensive arterial collateralization. Intra-operative assessment of the hepatic artery with trial clamping of the GDA is performed with Doppler ultrasound and palpation. Pulsation of the hepatic artery should remain consistent with respiration in the setting of successful management of MALS. Absent or inadequate flow of the hepatic artery with the above maneuverer warrants vascular reconstruction before ligating the GDA. Post-operatively, patients should be followed up by vascular surgery during the short-to-medium term to assess any changes in vascular anatomy on surveillance imaging. Rather than serving as a selection tool for MALS release, our algorithm aims to underscore the importance of case-by-case discussion in managing MALS in HPB patients and the mandatory steps in the decision-making process.

Upon reviewing the literature, we have identified significant gaps in the available research. For this high-risk patient population, several key questions remain unanswered: (1) Does endovascular stenting prior to radical HPB surgery improve patient outcomes? (2) Does the hybrid approach combining laparoscopic MAL release and endovascular intervention provide better outcomes compared to laparoscopic release alone in the general population? (3) Is the two-staged intervention superior to concurrent MAL release with HPB surgery? (4) Considering the risks of MAL release and endovascular stenting, what would be the optimal criteria for selecting low-risk HPB patients who would benefit from the expectant management of MALS? (5) How can we effectively stratify MALS patients who require complex vascular reconstruction during PD or OLT? Given that the current literature mainly consists of case reports, management of MALS in HPB patients is likely to vary among surgeons and different centers. This presents an opportunity for larger centers to conduct comprehensive, prospective, registry-based, or interventional studies.

## 5. Conclusions

In conclusion, HPB patients with MALS require thorough preoperative assessment and surgical planning. At our center, the majority of these patients undergo concurrent MAL release and HPB surgery. Intraoperative assessment of arterial flow in the hepatic artery and celiac trunk is crucial for all patients with MALS. Postoperatively, a high suspicion of ischemia should be maintained regardless of the chosen management approach. It is evident that there is a lack of clear evidence regarding the optimal management algorithm for these patients.

## 6. Search Strategies

We conducted an extensive literature search on PubMed MEDLINE, Scopus, and ClinicalTrials.gov using a combination of controlled keywords: “median arcuate ligament”, “celiac trunk”, “pancreatoduodenectomy”, “Whipple”, and “liver transplant”. We identified additional studies manually from pertinent studies. Only manuscripts published in English were included in the review. The screening of titles and abstracts, followed by full-text screening, was conducted by authors L.Y.L. and J.G.E.

## Abbreviations

GDA	gastroduodenal artery
HPB	Hepato-pancreato-biliary
MAL	median arcuate ligament
MALS	median arcuate ligament syndrome
PD	pancreaticoduodenectomy
SMA	superior mesenteric artery
